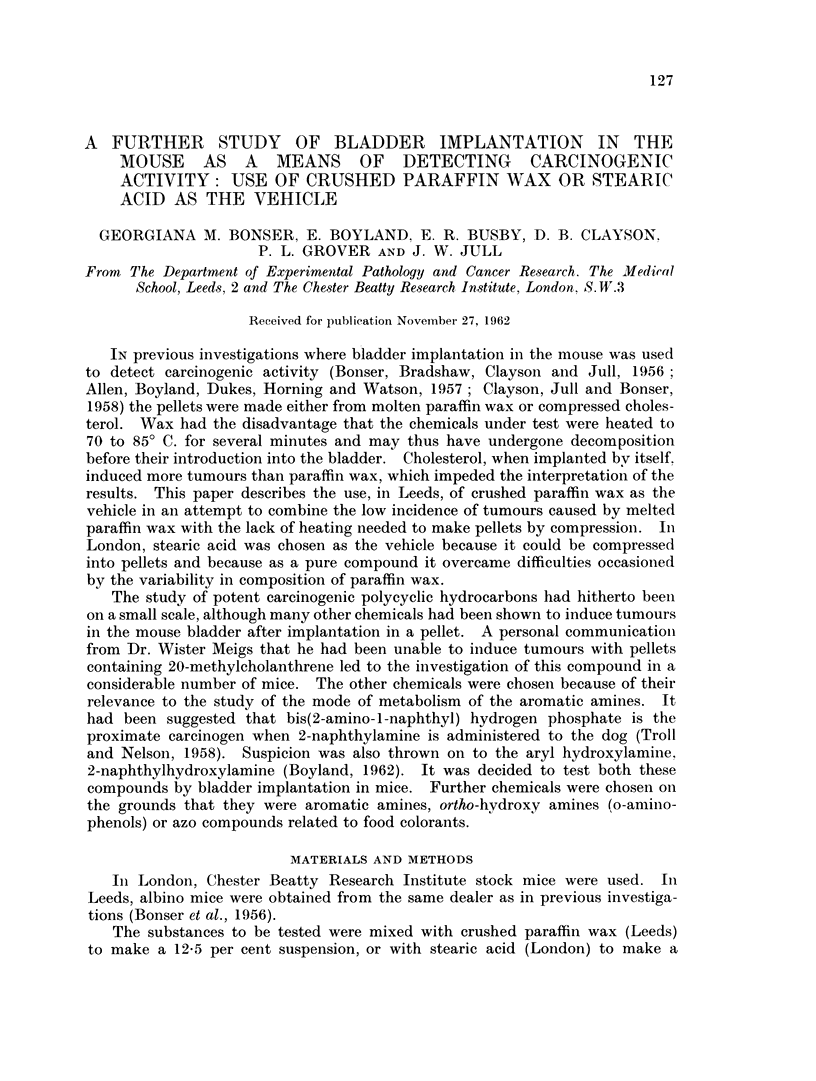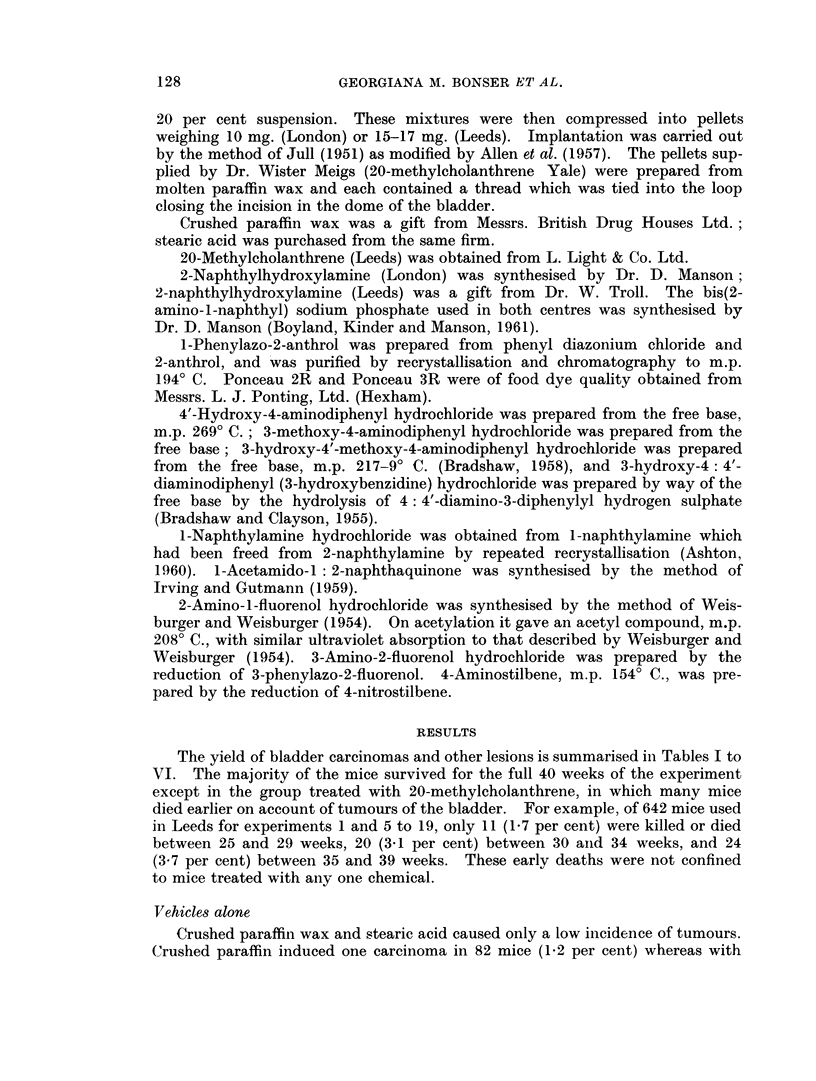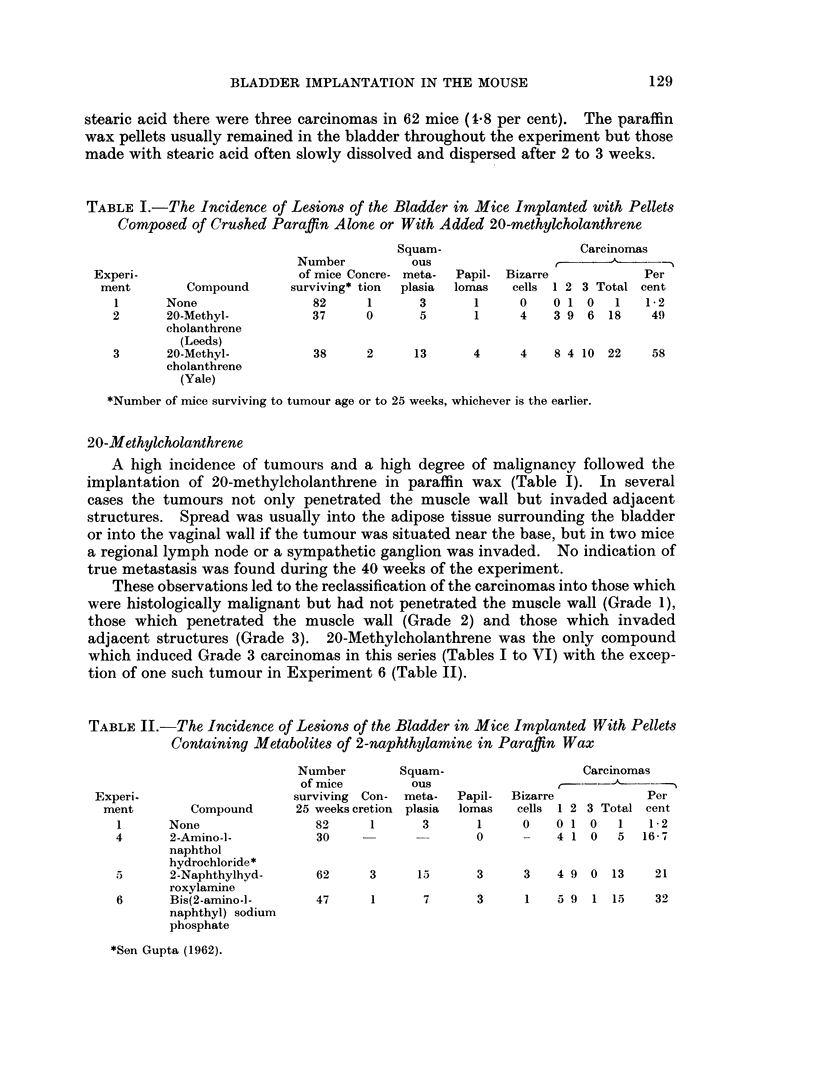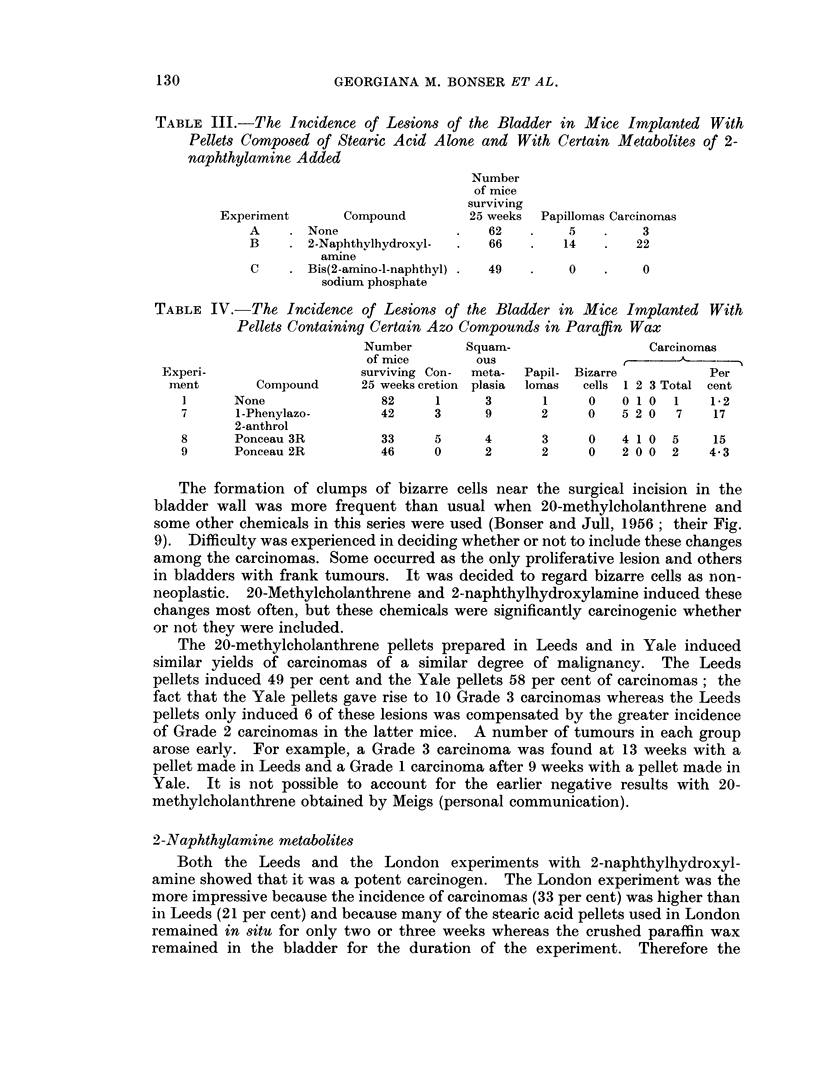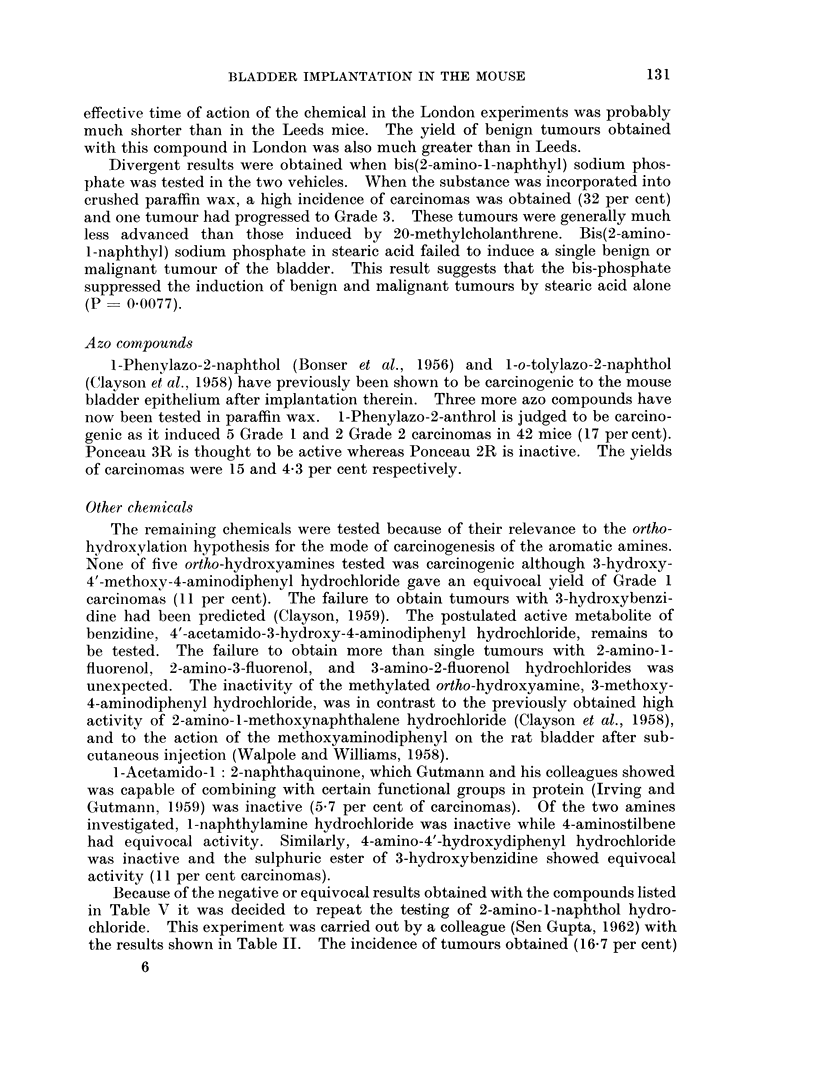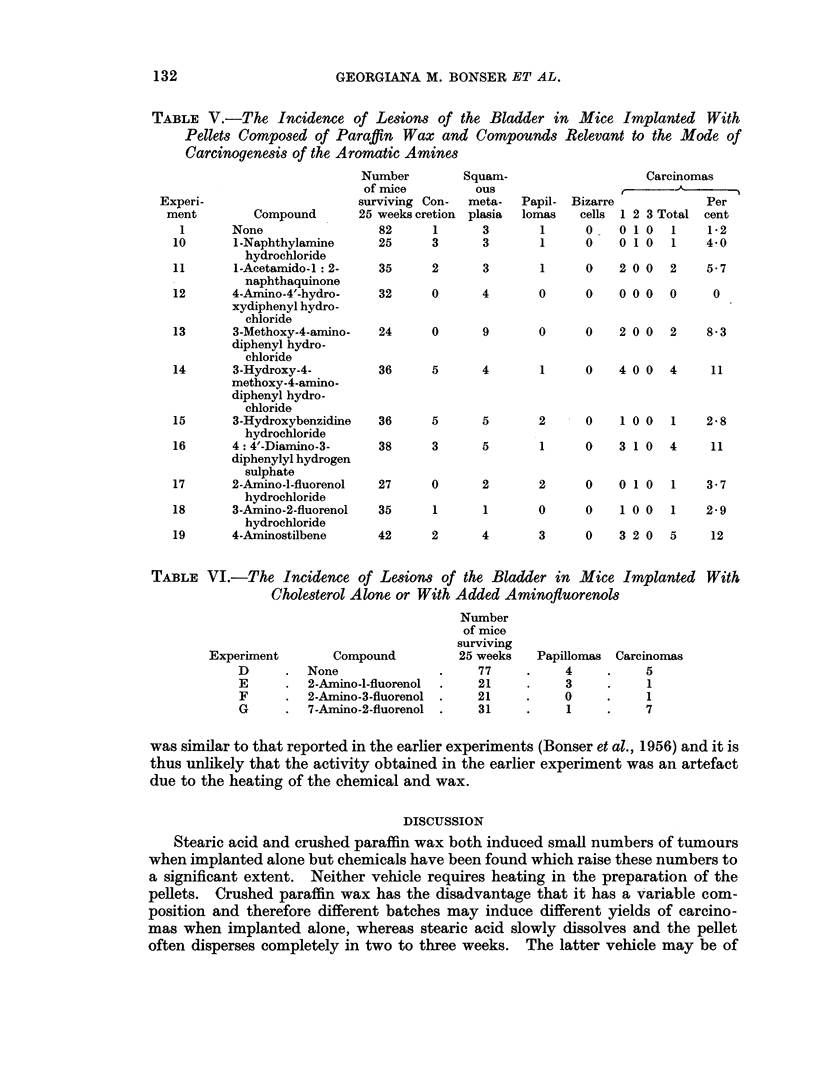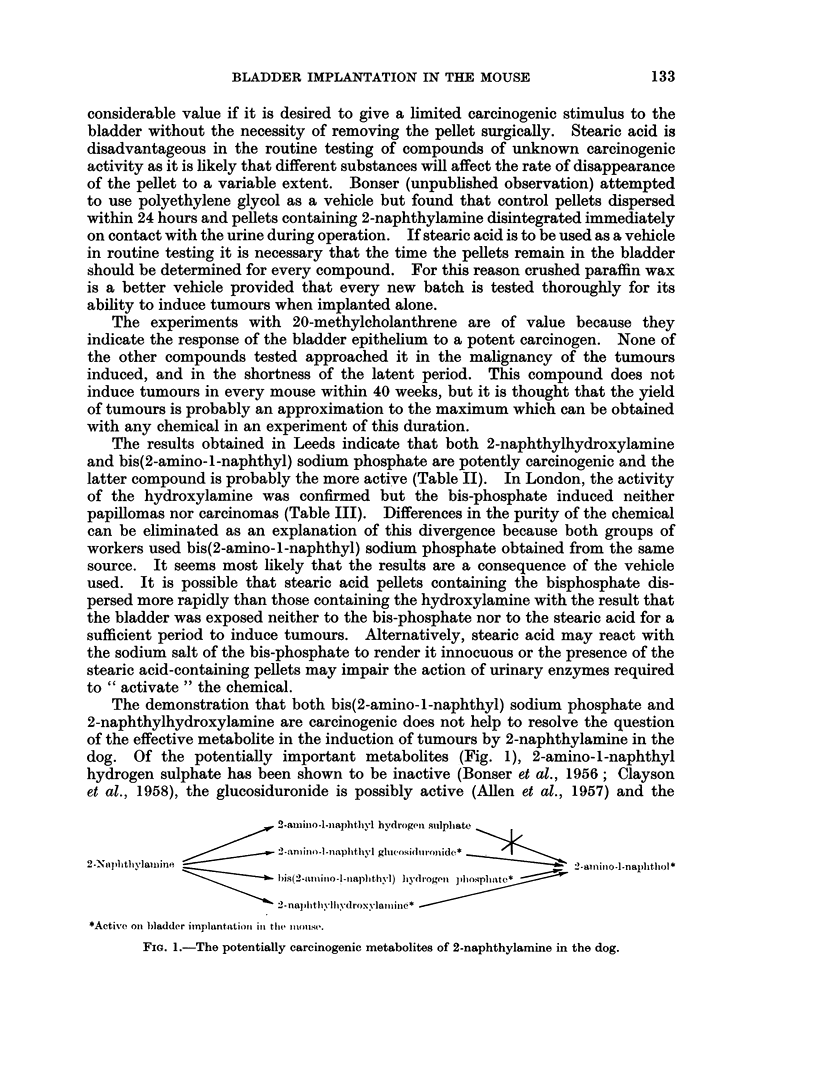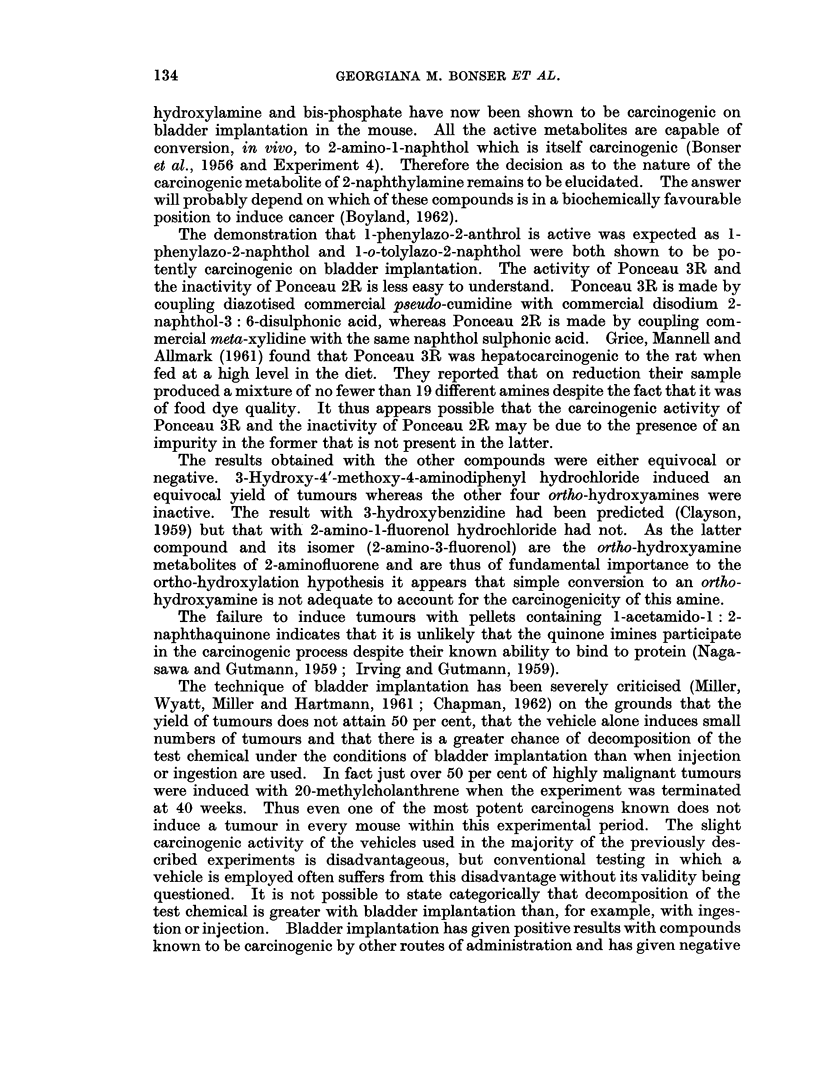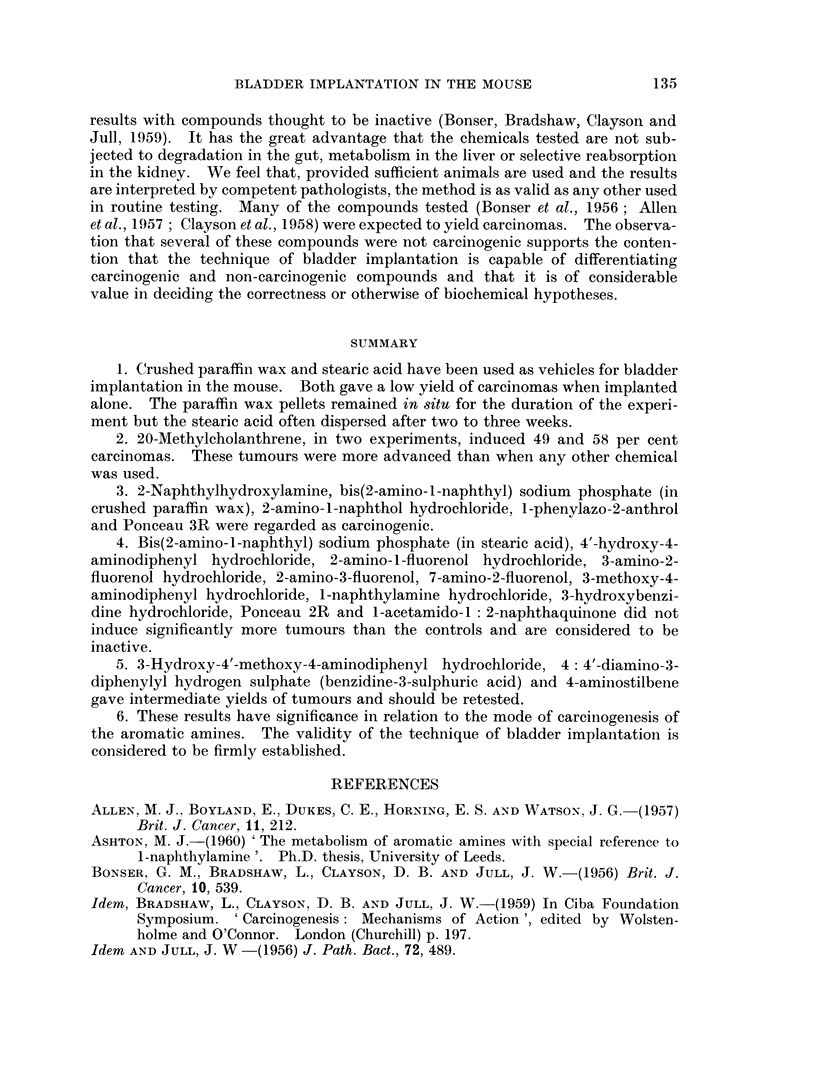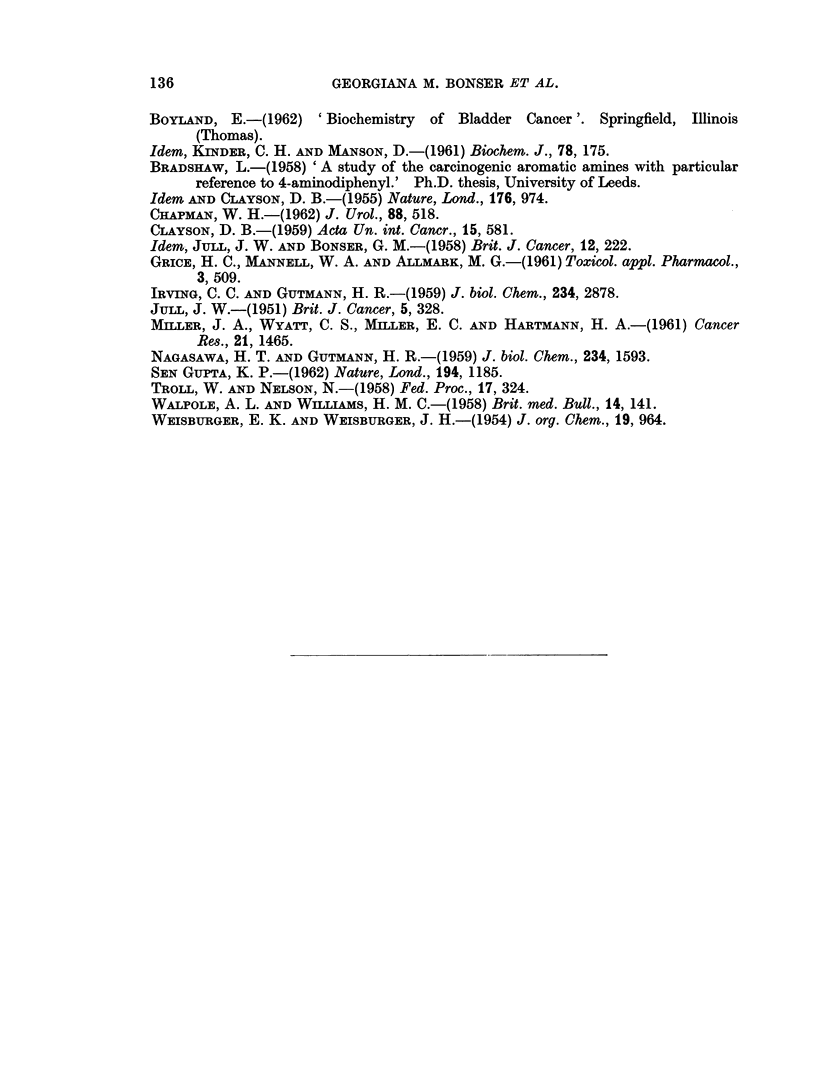# A Further Study of Bladder Implantation in the Mouse as a means of Detecting Carcinogenic Activity: Use of Crushed Paraffin Wax or Stearic Acid as the Vehicle

**DOI:** 10.1038/bjc.1963.19

**Published:** 1963-03

**Authors:** Georgiana M. Bonser, E. Boyland, E. R. Busby, D. B. Clayson, P. L. Grover, J. W. Jull


### A FURTHER STUDY OF BLADDER IMPLANTATION IN THE MOUSE AS A MEANS OF DETECTING CARCINOGENIC ACTIVITY: USE OF CRUSHED PARAFFIN WAX OR STEARIC ACID AS THE VEHICLE

GEORGIANA M. BONSER, E. BOYLAND, E. R. BUSBY, D. B. CLAYSON, P. L. GROVER AND J. W. JULL

*From The Department of Experimental Pathology and Cancer Research, The Medical School, Leeds, 2 and The Chester Beatty Research Institute, London, S.W.3*

Received for publication November 27, 1962

IN previous investigations where bladder implantation in the mouse was used to detect carcinogenic activity (Bonser, Bradshaw, Clayson and Jull, 1956; Allen, Boyland, Dukes, Horning and Watson, 1957; Clayson, Jull and Bonser, 1958) the pellets were made either from molten paraffin wax or compressed cholesterol. Wax had the disadvantage that the chemicals under test were heated to 70 to 85° C. for several minutes and may thus have undergone decomposition before their introduction into the bladder. Cholesterol, when implanted by itself, induced more tumours than paraffin wax, which impeded the interpretation of the results. This paper describes the use, in Leeds, of crushed paraffin wax as the vehicle in an attempt to combine the low incidence of tumours caused by melted paraffin wax with the lack of heating needed to make pellets by compression. In London, stearic acid was chosen as the vehicle because it could be compressed into pellets and because as a pure compound it overcame difficulties occasioned by the variability in composition of paraffin wax.

The study of potent carcinogenic polycyclic hydrocarbons had hitherto been on a small scale, although many other chemicals had been shown to induce tumours in the mouse bladder after implantation in a pellet. A personal communication from Dr. Wister Meigs that he had been unable to induce tumours with pellets containing 20-methylcholanthrene led to the investigation of this compound in a considerable number of mice. The other chemicals were chosen because of their relevance to the study of the mode of metabolism of the aromatic amines. It had been suggested that bis(2-amino-1-naphthyl) hydrogen phosphate is the proximate carcinogen when 2-naphthylamine is administered to the dog (Troll and Nelson, 1958). Suspicion was also thrown on to the aryl hydroxylamine, 2-naphthylhydroxylamine (Boyland, 1962). It was decided to test both these compounds by bladder implantation in mice. Further chemicals were chosen on the grounds that they were aromatic amines, *ortho*-hydroxy amines (o-aminophenols) or azo compounds related to food colorants.

### MATERIALS AND METHODS

In London, Chester Beatty Research Institute stock mice were used. In Leeds, albino mice were obtained from the same dealer as in previous investigations (Bonser *et al.*, 1956).

The substances to be tested were mixed with crushed paraffin wax (Leeds) to make a 12·5 per cent suspension, or with stearic acid (London) to make a

20 per cent suspension. These mixtures were then compressed into pellets weighing 10 mg. (London) or 15–17 mg. (Leeds). Implantation was carried out by the method of Jull (1951) as modified by Allen *et al.* (1957). The pellets supplied by Dr. Wister Meigs (20-methylcholanthrene Yale) were prepared from molten paraffin wax and each contained a thread which was tied into the loop closing the incision in the dome of the bladder.

Crushed paraffin wax was a gift from Messrs. British Drug Houses Ltd.; stearic acid was purchased from the same firm.

20-Methylcholanthrene (Leeds) was obtained from L. Light & Co. Ltd.

2-Naphthylhydroxylamine (London) was synthesised by Dr. D. Manson; 2-naphthylhydroxylamine (Leeds) was a gift from Dr. W. Troll. The bis(2-amino-1-naphthyl) sodium phosphate used in both centres was synthesised by Dr. D. Manson (Boyland, Kinder and Manson, 1961).

1-Phenylazo-2-anthrol was prepared from phenyl diazonium chloride and 2-anthrol, and was purified by recrystallisation and chromatography to m.p. 194° C. Ponceau 2R and Ponceau 3R were of food dye quality obtained from Messrs. L. J. Ponting, Ltd. (Hexham).

4′-Hydroxy-4-aminodiphenyl hydrochloride was prepared from the free base, m.p. 269° C.; 3-methoxy-4-aminodiphenyl hydrochloride was prepared from the free base; 3-hydroxy-4′-methoxy-4-aminodiphenyl hydrochloride was prepared from the free base, m.p. 217–9° C. (Bradshaw, 1958), and 3-hydroxy-4 : 4′-diaminodiphenyl (3-hydroxybenzidine) hydrochloride was prepared by way of the free base by the hydrolysis of 4 : 4′-diamino-3-diphenylyl hydrogen sulphate (Bradshaw and Clayson, 1955).

1-Naphthylamine hydrochloride was obtained from 1-naphthylamine which had been freed from 2-naphthylamine by repeated recrystallisation (Ashton, 1960). 1-Acetamido-1 : 2-naphthaquinone was synthesised by the method of Irving and Gutmann (1959).

2-Amino-1-fluorenol hydrochloride was synthesised by the method of Weisburger and Weisburger (1954). On acetylation it gave an acetyl compound, m.p. 208° C., with similar ultraviolet absorption to that described by Weisburger and Weisburger (1954). 3-Amino-2-fluorenol hydrochloride was prepared by the reduction of 3-phenylazo-2-fluorenol. 4-Aminostilbene, m.p. 154° C., was prepared by the reduction of 4-nitrostilbene.

### RESULTS

The yield of bladder carcinomas and other lesions is summarised in Tables I to VI. The majority of the mice survived for the full 40 weeks of the experiment except in the group treated with 20-methylcholanthrene, in which many mice died earlier on account of tumours of the bladder. For example, of 642 mice used in Leeds for experiments 1 and 5 to 19, only 11 (1·7 per cent) were killed or died between 25 and 29 weeks, 20 (3·1 per cent) between 30 and 34 weeks, and 24 (3·7 per cent) between 35 and 39 weeks. These early deaths were not confined to mice treated with any one chemical.

### *Vehicles alone*

Crushed paraffin wax and stearic acid caused only a low incidence of tumours. Crushed paraffin induced one carcinoma in 82 mice (1·2 per cent) whereas with stearic acid there were three carcinomas in 62 mice (4·8 per cent). The paraffin wax pellets usually remained in the bladder throughout the experiment but those made with stearic acid often slowly dissolved and dispersed after 2 to 3 weeks.

TABLE I.—*The Incidence of Lesions of the Bladder in Mice Implanted with Pellets Composed of Crushed Paraffin Alone or With Added* 20-*methylcholanthrene*

| Experiment | Compound | Number of mice surviving* | Concretion | Squamous metaplasia | Papillomas | Bizarre cells | Carcinomas 1 | 2 | 3 | Total | Per cent |
|---|---|---|---|---|---|---|---|---|---|---|---|
| 1 | None | 82 | 1 | 3 | 1 | 0 | 0 | 1 | 0 | 1 | 1·2 |
| 2 | 20-Methylcholanthrene (Leeds) | 37 | 0 | 5 | 1 | 4 | 3 | 9 | 6 | 18 | 49 |
| 3 | 20-Methylcholanthrene (Yale) | 38 | 2 | 13 | 4 | 4 | 8 | 4 | 10 | 22 | 58 |

*Number of mice surviving to tumour age or to 25 weeks, whichever is the earlier.

20-*Methylcholanthrene*

A high incidence of tumours and a high degree of malignancy followed the implantation of 20-methylcholanthrene in paraffin wax (Table I). In several cases the tumours not only penetrated the muscle wall but invaded adjacent structures. Spread was usually into the adipose tissue surrounding the bladder or into the vaginal wall if the tumour was situated near the base, but in two mice a regional lymph node or a sympathetic ganglion was invaded. No indication of true metastasis was found during the 40 weeks of the experiment.

These observations led to the reclassification of the carcinomas into those which were histologically malignant but had not penetrated the muscle wall (Grade 1), those which penetrated the muscle wall (Grade 2) and those which invaded adjacent structures (Grade 3). 20-Methylcholanthrene was the only compound which induced Grade 3 carcinomas in this series (Tables I to VI) with the exception of one such tumour in Experiment 6 (Table II).

TABLE II.—*The Incidence of Lesions of the Bladder in Mice Implanted With Pellets Containing Metabolites of 2-naphthylamine in Paraffin Wax*

| Experiment | Compound | Number of mice surviving 25 weeks | Concretion | Squamous metaplasia | Papillomas | Bizarre cells | Carcinomas 1 | 2 | 3 | Total | Per cent |
|---|---|---|---|---|---|---|---|---|---|---|---|
| 1 | None | 82 | 1 | 3 | 1 | 0 | 0 | 1 | 0 | 1 | 1·2 |
| 4 | 2-Amino-1-naphthol hydrochloride* | 30 | — | — | 0 | – | 4 | 1 | 0 | 5 | 16·7 |
| 5 | 2-Naphthylhydroxylamine | 62 | 3 | 15 | 3 | 3 | 4 | 9 | 0 | 13 | 21 |
| 6 | Bis(2-amino-1-naphthyl) sodium phosphate | 47 | 1 | 7 | 3 | 1 | 5 | 9 | 1 | 15 | 32 |

*Sen Gupta (1962).

TABLE III.—*The Incidence of Lesions of the Bladder in Mice Implanted With Pellets Composed of Stearic Acid Alone and With Certain Metabolites of 2-naphthylamine Added*

| Experiment | Compound | Number of mice surviving 25 weeks | Papillomas | Carcinomas |
|---|---|---|---|---|
| A | None | 62 | 5 | 3 |
| B | 2-Naphthylhydroxyl-amine | 66 | 14 | 22 |
| C | Bis(2-amino-l-naphthyl) sodium phosphate | 49 | 0 | 0 |

TABLE IV.—*The Incidence of Lesions of the Bladder in Mice Implanted With Pellets Containing Certain Azo Compounds in Paraffin Wax*

| Experi-ment | Compound | Number of mice surviving 25 weeks | Con-cretion | Squam-ous meta-plasia | Papil-lomas | Bizarre cells | Carcinomas 1 | 2 | 3 | Total | Per cent |
|---|---|---|---|---|---|---|---|---|---|---|---|
| 1 | None | 82 | 1 | 3 | 1 | 0 | 0 | 1 | 0 | 1 | 1·2 |
| 7 | 1-Phenylazo-2-anthrol | 42 | 3 | 9 | 2 | 0 | 5 | 2 | 0 | 7 | 17 |
| 8 | Ponceau 3R | 33 | 5 | 4 | 3 | 0 | 4 | 1 | 0 | 5 | 15 |
| 9 | Ponceau 2R | 46 | 0 | 2 | 2 | 0 | 2 | 0 | 0 | 2 | 4·3 |

The formation of clumps of bizarre cells near the surgical incision in the bladder wall was more frequent than usual when 20-methylcholanthrene and some other chemicals in this series were used (Bonser and Jull, 1956 ; their Fig. 9). Difficulty was experienced in deciding whether or not to include these changes among the carcinomas. Some occurred as the only proliferative lesion and others in bladders with frank tumours. It was decided to regard bizarre cells as non-neoplastic. 20-Methylcholanthrene and 2-naphthylhydroxylamine induced these changes most often, but these chemicals were significantly carcinogenic whether or not they were included.

The 20-methylcholanthrene pellets prepared in Leeds and in Yale induced similar yields of carcinomas of a similar degree of malignancy. The Leeds pellets induced 49 per cent and the Yale pellets 58 per cent of carcinomas ; the fact that the Yale pellets gave rise to 10 Grade 3 carcinomas whereas the Leeds pellets only induced 6 of these lesions was compensated by the greater incidence of Grade 2 carcinomas in the latter mice. A number of tumours in each group arose early. For example, a Grade 3 carcinoma was found at 13 weeks with a pellet made in Leeds and a Grade 1 carcinoma after 9 weeks with a pellet made in Yale. It is not possible to account for the earlier negative results with 20-methylcholanthrene obtained by Meigs (personal communication).

### 2-*Naphthylamine metabolites*

Both the Leeds and the London experiments with 2-naphthylhydroxylamine showed that it was a potent carcinogen. The London experiment was the more impressive because the incidence of carcinomas (33 per cent) was higher than in Leeds (21 per cent) and because many of the stearic acid pellets used in London remained *in situ* for only two or three weeks whereas the crushed paraffin wax remained in the bladder for the duration of the experiment. Therefore the effective time of action of the chemical in the London experiments was probably much shorter than in the Leeds mice. The yield of benign tumours obtained with this compound in London was also much greater than in Leeds.

Divergent results were obtained when bis(2-amino-1-naphthyl) sodium phosphate was tested in the two vehicles. When the substance was incorporated into crushed paraffin wax, a high incidence of carcinomas was obtained (32 per cent) and one tumour had progressed to Grade 3. These tumours were generally much less advanced than those induced by 20-methylcholanthrene. Bis(2-amino-1-naphthyl) sodium phosphate in stearic acid failed to induce a single benign or malignant tumour of the bladder. This result suggests that the bis-phosphate suppressed the induction of benign and malignant tumours by stearic acid alone ($P = 0{\cdot}0077$).

*Azo compounds*

1-Phenylazo-2-naphthol (Bonser *et al.*, 1956) and 1-*o*-tolylazo-2-naphthol (Clayson *et al.*, 1958) have previously been shown to be carcinogenic to the mouse bladder epithelium after implantation therein. Three more azo compounds have now been tested in paraffin wax. 1-Phenylazo-2-anthrol is judged to be carcinogenic as it induced 5 Grade 1 and 2 Grade 2 carcinomas in 42 mice (17 per cent). Ponceau 3R is thought to be active whereas Ponceau 2R is inactive. The yields of carcinomas were 15 and 4·3 per cent respectively.

*Other chemicals*

The remaining chemicals were tested because of their relevance to the *ortho*-hydroxylation hypothesis for the mode of carcinogenesis of the aromatic amines. None of five *ortho*-hydroxyamines tested was carcinogenic although 3-hydroxy-4′-methoxy-4-aminodiphenyl hydrochloride gave an equivocal yield of Grade 1 carcinomas (11 per cent). The failure to obtain tumours with 3-hydroxybenzidine had been predicted (Clayson, 1959). The postulated active metabolite of benzidine, 4′-acetamido-3-hydroxy-4-aminodiphenyl hydrochloride, remains to be tested. The failure to obtain more than single tumours with 2-amino-1-fluorenol, 2-amino-3-fluorenol, and 3-amino-2-fluorenol hydrochlorides was unexpected. The inactivity of the methylated *ortho*-hydroxyamine, 3-methoxy-4-aminodiphenyl hydrochloride, was in contrast to the previously obtained high activity of 2-amino-1-methoxynaphthalene hydrochloride (Clayson *et al.*, 1958), and to the action of the methoxyaminodiphenyl on the rat bladder after subcutaneous injection (Walpole and Williams, 1958).

1-Acetamido-1 : 2-naphthaquinone, which Gutmann and his colleagues showed was capable of combining with certain functional groups in protein (Irving and Gutmann, 1959) was inactive (5·7 per cent of carcinomas). Of the two amines investigated, 1-naphthylamine hydrochloride was inactive while 4-aminostilbene had equivocal activity. Similarly, 4-amino-4′-hydroxydiphenyl hydrochloride was inactive and the sulphuric ester of 3-hydroxybenzidine showed equivocal activity (11 per cent carcinomas).

Because of the negative or equivocal results obtained with the compounds listed in Table V it was decided to repeat the testing of 2-amino-1-naphthol hydrochloride. This experiment was carried out by a colleague (Sen Gupta, 1962) with the results shown in Table II. The incidence of tumours obtained (16·7 per cent)

TABLE V.—*The Incidence of Lesions of the Bladder in Mice Implanted With Pellets Composed of Paraffin Wax and Compounds Relevant to the Mode of Carcinogenesis of the Aromatic Amines*

| Experiment | Compound | Number of mice surviving 25 weeks | Concretion | Squamous metaplasia | Papillomas | Bizarre cells | Carcinomas 1 | 2 | 3 | Total | Per cent |
|---|---|---|---|---|---|---|---|---|---|---|---|
| 1 | None | 82 | 1 | 3 | 1 | 0 | 0 | 1 | 0 | 1 | 1·2 |
| 10 | 1-Naphthylamine hydrochloride | 25 | 3 | 3 | 1 | 0 | 0 | 1 | 0 | 1 | 4·0 |
| 11 | 1-Acetamido-1 : 2-naphthaquinone | 35 | 2 | 3 | 1 | 0 | 2 | 0 | 0 | 2 | 5·7 |
| 12 | 4-Amino-4′-hydroxydiphenyl hydrochloride | 32 | 0 | 4 | 0 | 0 | 0 | 0 | 0 | 0 | 0 |
| 13 | 3-Methoxy-4-aminodiphenyl hydrochloride | 24 | 0 | 9 | 0 | 0 | 2 | 0 | 0 | 2 | 8·3 |
| 14 | 3-Hydroxy-4-methoxy-4-aminodiphenyl hydrochloride | 36 | 5 | 4 | 1 | 0 | 4 | 0 | 0 | 4 | 11 |
| 15 | 3-Hydroxybenzidine hydrochloride | 36 | 5 | 5 | 2 | 0 | 1 | 0 | 0 | 1 | 2·8 |
| 16 | 4 : 4′-Diamino-3-diphenylyl hydrogen sulphate | 38 | 3 | 5 | 1 | 0 | 3 | 1 | 0 | 4 | 11 |
| 17 | 2-Amino-1-fluorenol hydrochloride | 27 | 0 | 2 | 2 | 0 | 0 | 1 | 0 | 1 | 3·7 |
| 18 | 3-Amino-2-fluorenol hydrochloride | 35 | 1 | 1 | 0 | 0 | 1 | 0 | 0 | 1 | 2·9 |
| 19 | 4-Aminostilbene | 42 | 2 | 4 | 3 | 0 | 3 | 2 | 0 | 5 | 12 |

TABLE VI.—*The Incidence of Lesions of the Bladder in Mice Implanted With Cholesterol Alone or With Added Aminofluorenols*

| Experiment | Compound | Number of mice surviving 25 weeks | Papillomas | Carcinomas |
|---|---|---|---|---|
| D | None | 77 | 4 | 5 |
| E | 2-Amino-1-fluorenol | 21 | 3 | 1 |
| F | 2-Amino-3-fluorenol | 21 | 0 | 1 |
| G | 7-Amino-2-fluorenol | 31 | 1 | 7 |

was similar to that reported in the earlier experiments (Bonser *et al.*, 1956) and it is thus unlikely that the activity obtained in the earlier experiment was an artefact due to the heating of the chemical and wax.

### DISCUSSION

Stearic acid and crushed paraffin wax both induced small numbers of tumours when implanted alone but chemicals have been found which raise these numbers to a significant extent. Neither vehicle requires heating in the preparation of the pellets. Crushed paraffin wax has the disadvantage that it has a variable composition and therefore different batches may induce different yields of carcinomas when implanted alone, whereas stearic acid slowly dissolves and the pellet often disperses completely in two to three weeks. The latter vehicle may be of considerable value if it is desired to give a limited carcinogenic stimulus to the bladder without the necessity of removing the pellet surgically. Stearic acid is disadvantageous in the routine testing of compounds of unknown carcinogenic activity as it is likely that different substances will affect the rate of disappearance of the pellet to a variable extent. Bonser (unpublished observation) attempted to use polyethylene glycol as a vehicle but found that control pellets dispersed within 24 hours and pellets containing 2-naphthylamine disintegrated immediately on contact with the urine during operation. If stearic acid is to be used as a vehicle in routine testing it is necessary that the time the pellets remain in the bladder should be determined for every compound. For this reason crushed paraffin wax is a better vehicle provided that every new batch is tested thoroughly for its ability to induce tumours when implanted alone.

The experiments with 20-methylcholanthrene are of value because they indicate the response of the bladder epithelium to a potent carcinogen. None of the other compounds tested approached it in the malignancy of the tumours induced, and in the shortness of the latent period. This compound does not induce tumours in every mouse within 40 weeks, but it is thought that the yield of tumours is probably an approximation to the maximum which can be obtained with any chemical in an experiment of this duration.

The results obtained in Leeds indicate that both 2-naphthylhydroxylamine and bis(2-amino-1-naphthyl) sodium phosphate are potently carcinogenic and the latter compound is probably the more active (Table II). In London, the activity of the hydroxylamine was confirmed but the bis-phosphate induced neither papillomas nor carcinomas (Table III). Differences in the purity of the chemical can be eliminated as an explanation of this divergence because both groups of workers used bis(2-amino-1-naphthyl) sodium phosphate obtained from the same source. It seems most likely that the results are a consequence of the vehicle used. It is possible that stearic acid pellets containing the bisphosphate dispersed more rapidly than those containing the hydroxylamine with the result that the bladder was exposed neither to the bis-phosphate nor to the stearic acid for a sufficient period to induce tumours. Alternatively, stearic acid may react with the sodium salt of the bis-phosphate to render it innocuous or the presence of the stearic acid-containing pellets may impair the action of urinary enzymes required to " activate " the chemical.

The demonstration that both bis(2-amino-1-naphthyl) sodium phosphate and 2-naphthylhydroxylamine are carcinogenic does not help to resolve the question of the effective metabolite in the induction of tumours by 2-naphthylamine in the dog. Of the potentially important metabolites (Fig. 1), 2-amino-1-naphthyl hydrogen sulphate has been shown to be inactive (Bonser *et al.*, 1956 ; Clayson *et al.*, 1958), the glucosiduronide is possibly active (Allen *et al.*, 1957) and the

FIG. 1.—The potentially carcinogenic metabolites of 2-naphthylamine in the dog.

hydroxylamine and bis-phosphate have now been shown to be carcinogenic on bladder implantation in the mouse. All the active metabolites are capable of conversion, *in vivo*, to 2-amino-1-naphthol which is itself carcinogenic (Bonser *et al.*, 1956 and Experiment 4). Therefore the decision as to the nature of the carcinogenic metabolite of 2-naphthylamine remains to be elucidated. The answer will probably depend on which of these compounds is in a biochemically favourable position to induce cancer (Boyland, 1962).

The demonstration that 1-phenylazo-2-anthrol is active was expected as 1-phenylazo-2-naphthol and 1-*o*-tolylazo-2-naphthol were both shown to be potently carcinogenic on bladder implantation. The activity of Ponceau 3R and the inactivity of Ponceau 2R is less easy to understand. Ponceau 3R is made by coupling diazotised commercial *pseudo*-cumidine with commercial disodium 2-naphthol-3 : 6-disulphonic acid, whereas Ponceau 2R is made by coupling commercial *meta*-xylidine with the same naphthol sulphonic acid. Grice, Mannell and Allmark (1961) found that Ponceau 3R was hepatocarcinogenic to the rat when fed at a high level in the diet. They reported that on reduction their sample produced a mixture of no fewer than 19 different amines despite the fact that it was of food dye quality. It thus appears possible that the carcinogenic activity of Ponceau 3R and the inactivity of Ponceau 2R may be due to the presence of an impurity in the former that is not present in the latter.

The results obtained with the other compounds were either equivocal or negative. 3-Hydroxy-4′-methoxy-4-aminodiphenyl hydrochloride induced an equivocal yield of tumours whereas the other four *ortho*-hydroxyamines were inactive. The result with 3-hydroxybenzidine had been predicted (Clayson, 1959) but that with 2-amino-1-fluorenol hydrochloride had not. As the latter compound and its isomer (2-amino-3-fluorenol) are the *ortho*-hydroxyamine metabolites of 2-aminofluorene and are thus of fundamental importance to the ortho-hydroxylation hypothesis it appears that simple conversion to an *ortho*-hydroxyamine is not adequate to account for the carcinogenicity of this amine.

The failure to induce tumours with pellets containing 1-acetamido-1 : 2-naphthaquinone indicates that it is unlikely that the quinone imines participate in the carcinogenic process despite their known ability to bind to protein (Nagasawa and Gutmann, 1959 ; Irving and Gutmann, 1959).

The technique of bladder implantation has been severely criticised (Miller, Wyatt, Miller and Hartmann, 1961 ; Chapman, 1962) on the grounds that the yield of tumours does not attain 50 per cent, that the vehicle alone induces small numbers of tumours and that there is a greater chance of decomposition of the test chemical under the conditions of bladder implantation than when injection or ingestion are used. In fact just over 50 per cent of highly malignant tumours were induced with 20-methylcholanthrene when the experiment was terminated at 40 weeks. Thus even one of the most potent carcinogens known does not induce a tumour in every mouse within this experimental period. The slight carcinogenic activity of the vehicles used in the majority of the previously described experiments is disadvantageous, but conventional testing in which a vehicle is employed often suffers from this disadvantage without its validity being questioned. It is not possible to state categorically that decomposition of the test chemical is greater with bladder implantation than, for example, with ingestion or injection. Bladder implantation has given positive results with compounds known to be carcinogenic by other routes of administration and has given negative results with compounds thought to be inactive (Bonser, Bradshaw, Clayson and Jull, 1959). It has the great advantage that the chemicals tested are not subjected to degradation in the gut, metabolism in the liver or selective reabsorption in the kidney. We feel that, provided sufficient animals are used and the results are interpreted by competent pathologists, the method is as valid as any other used in routine testing. Many of the compounds tested (Bonser *et al.*, 1956 ; Allen *et al.*, 1957 ; Clayson *et al.*, 1958) were expected to yield carcinomas. The observation that several of these compounds were not carcinogenic supports the contention that the technique of bladder implantation is capable of differentiating carcinogenic and non-carcinogenic compounds and that it is of considerable value in deciding the correctness or otherwise of biochemical hypotheses.

### SUMMARY

1. Crushed paraffin wax and stearic acid have been used as vehicles for bladder implantation in the mouse. Both gave a low yield of carcinomas when implanted alone. The paraffin wax pellets remained *in situ* for the duration of the experiment but the stearic acid often dispersed after two to three weeks.

2. 20-Methylcholanthrene, in two experiments, induced 49 and 58 per cent carcinomas. These tumours were more advanced than when any other chemical was used.

3. 2-Naphthylhydroxylamine, bis(2-amino-1-naphthyl) sodium phosphate (in crushed paraffin wax), 2-amino-1-naphthol hydrochloride, 1-phenylazo-2-anthrol and Ponceau 3R were regarded as carcinogenic.

4. Bis(2-amino-1-naphthyl) sodium phosphate (in stearic acid), 4′-hydroxy-4-aminodiphenyl hydrochloride, 2-amino-1-fluorenol hydrochloride, 3-amino-2-fluorenol hydrochloride, 2-amino-3-fluorenol, 7-amino-2-fluorenol, 3-methoxy-4-aminodiphenyl hydrochloride, 1-naphthylamine hydrochloride, 3-hydroxybenzidine hydrochloride, Ponceau 2R and 1-acetamido-1 : 2-naphthaquinone did not induce significantly more tumours than the controls and are considered to be inactive.

5. 3-Hydroxy-4′-methoxy-4-aminodiphenyl hydrochloride, 4 : 4′-diamino-3-diphenylyl hydrogen sulphate (benzidine-3-sulphuric acid) and 4-aminostilbene gave intermediate yields of tumours and should be retested.

6. These results have significance in relation to the mode of carcinogenesis of the aromatic amines. The validity of the technique of bladder implantation is considered to be firmly established.

### REFERENCES

ALLEN, M. J., BOYLAND, E., DUKES, C. E., HORNING, E. S. AND WATSON, J. G.—(1957) *Brit. J. Cancer*, **11**, 212.

ASHTON, M. J.—(1960) ‘ The metabolism of aromatic amines with special reference to 1-naphthylamine ’. Ph.D. thesis, University of Leeds.

BONSER, G. M., BRADSHAW, L., CLAYSON, D. B. AND JULL, J. W.—(1956) *Brit. J. Cancer*, **10**, 539.

*Idem*, BRADSHAW, L., CLAYSON, D. B. AND JULL, J. W.—(1959) In Ciba Foundation Symposium. ‘ Carcinogenesis : Mechanisms of Action ’, edited by Wolstenholme and O’Connor. London (Churchill) p. 197.

*Idem* AND JULL, J. W.—(1956) *J. Path. Bact.*, **72**, 489.

BOYLAND, E.—(1962) 'Biochemistry of Bladder Cancer'. Springfield, Illinois (Thomas).
*Idem*, KINDER, C. H. AND MANSON, D.—(1961) *Biochem. J.*, **78**, 175.
BRADSHAW, L.—(1958) 'A study of the carcinogenic aromatic amines with particular reference to 4-aminodiphenyl.' Ph.D. thesis, University of Leeds.
*Idem* AND CLAYSON, D. B.—(1955) *Nature, Lond.*, **176**, 974.
CHAPMAN, W. H.—(1962) *J. Urol.*, **88**, 518.
CLAYSON, D. B.—(1959) *Acta Un. int. Cancr.*, **15**, 581.
*Idem*, JULL, J. W. AND BONSER, G. M.—(1958) *Brit. J. Cancer*, **12**, 222.
GRICE, H. C., MANNELL, W. A. AND ALLMARK, M. G.—(1961) *Toxicol. appl. Pharmacol.*, **3**, 509.
IRVING, C. C. AND GUTMANN, H. R.—(1959) *J. biol. Chem.*, **234**, 2878.
JULL, J. W.—(1951) *Brit. J. Cancer*, **5**, 328.
MILLER, J. A., WYATT, C. S., MILLER, E. C. AND HARTMANN, H. A.—(1961) *Cancer Res.*, **21**, 1465.
NAGASAWA, H. T. AND GUTMANN, H. R.—(1959) *J. biol. Chem.*, **234**, 1593.
SEN GUPTA, K. P.—(1962) *Nature, Lond.*, **194**, 1185.
TROLL, W. AND NELSON, N.—(1958) *Fed. Proc.*, **17**, 324.
WALPOLE, A. L. AND WILLIAMS, H. M. C.—(1958) *Brit. med. Bull.*, **14**, 141.
WEISBURGER, E. K. AND WEISBURGER, J. H.—(1954) *J. org. Chem.*, **19**, 964.